# Hepatitis E rORF2p Stimulated and Unstimulated Peripheral Expression Profiling in Patients with Self-Limiting Hepatitis E Infection

**DOI:** 10.1155/2014/565284

**Published:** 2014-05-15

**Authors:** Sanjay B. Rathod, Anuradha S. Tripathy

**Affiliations:** Hepatitis Group, National Institute of Virology, Pune, 130/1, Sus Road, Pashan, Pune, Maharashtra 411021, India

## Abstract

To improve the current knowledge on the involvement of peripheral lymphocytes in hepatitis E virus (HEV) associated pathogenesis, we analyzed alterations in (1) immunophenotypic expressions (by flow cytometry) and (2) gene expression patterns (by TaqMan Low Density Array) of activatory, inhibitory, integrin, homing, ectonucleotidase machinery, costimulatory, inflammatory markers, and T regulatory cells (Treg) associated cytokines on HEV rORF2p stimulated and unstimulated PBMCs of 43 acute HEV patients, 30 recovered individuals, and 43 controls. The phenotypic expressions of key molecules CTLA-4, GITR, CD103, CD25, CD69, IL10 and TGF-**β**_1_ in the acute patients and TGF-**β**_1_ in the recovered individuals were significantly elevated on both unstimulated and stimulated PBMCs. Gene expression array data revealed upregulations of CD25, PD1, CD103, CCR4, IL10, and TGF-**β**_1_ on both unstimulated and HEV rORF2p stimulated PBMCs of acute patients. The observed upregulations of inhibitory, integrin, activatory, and Treg-associated cytokine genes on the PBMCs of acute HEV patients complemented by their frequency data suggest them as the major players in the fine-tuning of immune response in self-limiting hepatitis E infection.

## 1. Introduction

Hepatitis E virus (HEV), a fecoorally transmitted virus, the most common cause of enterically transmitted acute hepatitis in the developing countries [1], has emerged as an important issue in the developed countries over the past decade. However, the cases differ from those in developing countries in being possibly caused by zoonotic transmission, often affecting immunocompromised patients and occasionally leading to persistent HEV infection [2]. Reports from the Indian subcontinent indicating high mortality in patients with HEV have resulted in attempts to understand HEV pathogenesis. Understanding the immune correlates that contribute to the host immune response leading to recovery may help in designing an efficacious vaccine/immune based treatment strategy.

Reports have indicated that immune response to HEV infection underlies the pathogenesis of disease [3–7]. Complying this, there are reports indicating the involvement of cytokines, chemokines, and soluble factors in self-limiting HEV infection [5–9]. However, up/downregulation of cytokines and soluble receptors is an indirect measurement of immune activation not revealing which immune cells are activated. Reports have suggested that CD4^+^ T cells may have a direct effect on viral infections* in vivo* [10–12].

During infection, antigen-specific lymphocytes get activated and the activated T cells are responsible for T cell recruitment to the liver and for triggering of immune injury. In the same context,* in vitro* data on peripheral blood mononuclear cells (PBMCs) proliferation and HEV-specific T cells producing Th_2_ cytokine suggest a role of CD4^+^ T lymphocytes in HEV infection [5, 13]. Reports from our group and others suggest the involvement of both T cell subsets and antigen nonspecific cells in self-limiting HEV infection [6, 7, 13, 14]. We have recently reported peripheral CD11c, CD80, and CD83 expressions to be high in hepatitis E patients, CD11c expression to be positively associated with HEV replication [14], and association of T regulatory (Treg) cells in acute HEV infection [8]. Higher expressions of CTLA-4, PD1, GITR, CD95, CD103, and CD73 on T regulatory and T effector cells of HEV patients have indicated probable involvement of these molecules in Treg-mediated suppression [9].

To gain insight on how HEV infection influences the overall expression profiles on the PBMCs, we analyzed and compared the alterations in unstimulated and HEV rORF2p stimulated immunophenotypic expressions (by flow cytometry), and gene expression patterns (by TaqMan Low Density Array, TLDA) of activatory, inhibitory, homing, integrin, ectonucleotidase machinery, costimulatory, inflammatory markers, and Treg-associated cytokines in the PBMCs of patients with self-resolving HEV infection.

## 2. Material and Methods

### 2.1. Ethics Statement

This study was approved by the “Institutional Ethical Committee (IEC) for Research on Humans” as per the guidelines of Indian Council of Medical Research (ICMR). The participants had signed the informed consent form for use of their data in this particular study.

### 2.2. Study Population

Details of 116 individuals, including 43 patients in the acute phase of hepatitis E infection, 30 recovered individuals from hepatitis E, and 43 anti-HEV negative healthy controls enrolled in the study are depicted in Table 1. Classification of patients as acute and recovered individuals was done based on the standard clinical and biochemical criteria [5]. Briefly, patients presenting with icterus, dark-colored urine, elevated alanine aminotransferase (ALT) (normal level, 4–40 IU/L), and/or bilirubin levels (>1 mg/mL) in the serum, and/or presence of bile salts and pigments in the urine were considered to have acute hepatitis (AVH-E). All AVH-E patients had typical symptoms of acute viral hepatitis, such as sudden onset of fever, nausea, vomiting, weakness, and jaundice. Diagnosis of AVH-E was based on the presence of IgM antibodies to hepatitis E virus (IgM-anti-HEV) as detected by ELISA [15].The specificity of the assay (IgM anti HEV) was assessed using serum samples from 180 school children, the age group in which the disease is known to be less prevalent, and none was found positive indicating that the test was highly specific. Similarly, for assessment of sensitivity of the in-house kit, the results were compared with one commercially available kit that yielded a concordance of 85.6%. The recovered individuals having a recent history of acute hepatitis E had normalized ALT levels, positive for anti-HEV IgG antibody, and were positive/negative for serum anti-HEV IgM antibody. The control group consisted of age- and sex-matched apparently healthy individuals negative for HBsAg, anti-HIV, anti-HCV, IgM/IgG anti-HEV, and IgM anti-HAV antibodies and had the same epidemiological condition as patients. Thus, the control group was naïve to HEV infection. The patient population negative for HBsAg, anti-HIV, IgM anti-HAV, anti-HCV, and anti-HIV antibodies was only included in the study. None of the patients was having any past history of chronic liver disease and severe systemic illness or was undergoing therapy at the time of sampling. The patients as well as controls enrolled were from Western Maharashtra, India.

### 2.3. Serological and Molecular Testing

The samples were screened for the presence of IgM antibodies against hepatitis A virus (IgM anti-HAV; Hepavase A-96, General Biologicals Corporation, Hsin Chu, Taiwan), hepatitis B surface antigen (HBsAg; Surase B-96, General Biologicals Corporation, Hsin Chu, Taiwan), IgM antibodies to hepatitis B core antigen (IgM anti-HBc; Anticorase B-96, General Biologicals Corporation, Hsin Chu, Taiwan), antibodies to hepatitis C (anti-HCV; Ortho HCV 3.0, Ortho Clinical Diagnostics, NJ, USA), antibodies to HIV-1 (INSTI HIV-1 antibody Test Kit, Biological Laboratories Inc., Richmond, Canada), IgM and IgG anti-HEV antibodies by ELISA based on the use of recombinant ORF2 antigen (rORF2p) [15], and alanine aminotransferase levels (ALT; Span Diagnostics, Gujarat, India).

### 2.4. Preparation of Recombinant ORF2 Protein (rORF2p)

Complete* ORF2* gene (1983bp: 5147-7129ntd, corresponding to 660aa) from genotype 1 of HEV was cloned in pFastBac1 vector using Bac-to-Bac baculovirus expression system. Briefly, complete* ORF2* was amplified from the viral RNA extracted from a HEV RNA positive human fecal sample collected from an outbreak. TA cloning was done using pMosBlueT vector. This construct was then transformed and the insert was further cloned in pFastBac vector and this construct was transformed for generation of recombinant bacmids. The recombinant bacmids were transfected into Sf9 insect cells for preparation of high titer viral stock that was used further for expression of rORF2p in Sf9 cells. The protein was purified by anion exchange HPLC as reported by us [16].

### 2.5. Preparation of PBMCs and Experimental Design

PBMCs isolated by Ficoll-Hypaque (Sigma, USA) density gradient centrifugation method from the blood samples collected in K_3_-EDTA tube were resuspended in RPMI-1640 medium (Life Technologies, CA USA), supplemented with 2 mmol/L L-Glutamine, 1 mmol/L sodium pyruvate and 20 ug/mL of gentamycin (Sigma, USA). The viability of the cells was >95% as assessed by staining with 0.1% Trypan blue in PBS (Life Technologies, CA USA). Fresh PBMCs were used for staining followed by acquisition and analysis by flow cytometry. Part of the PBMCs was frozen to be used for assessing the gene expression profiles by custom made TaqMan Low Density Array (TLDA) system (Life Technologies, CA USA).

PBMCs stimulated with 10 ug/mL of purified rORF2p were cultured at 37°C under 5% CO_2_. Unstimulated PBMCs served as control. Cells harvested at 72 hr were assessed for the expression patterns of immune cell markers by flow cytometry and by TLDA.

### 2.6. Peripheral Immune Cell Marker Frequencies by Flow Cytometry

Anti-human monoclonal antibodies specific for apoptotic/inhibitory molecules (CD95 (Fas/Apo1), CD152 (CTLA-4), CD279 (PD1), CD274 (PD1-L), and CD357 (GITR)), activation molecules (CD25 (sIL2R*α*), CD69, CD71, and HLA-DR), chemokine and homing receptors (CD194 (CCR4), CD197 (CCR7), and CD62L (L-selectin)), integrin (CD103 (*α*E*β*7)), ectonucleotidase (CD39 (ENTPD1), CD73 (5′NT)),* naïve* (CD45RA), costimulatory molecules (CD40L (CD154), CD28, CD40, CD70, CD278(ICOS), and CD137 (4-1BB)), and immunoregulatory cytokines (IL10, TGF-**β**_1_) were used. Except for GITR (Miltenyi Biotec, Germany) and IL10 and TGF-**β**_1_ (eBioscience San Diego, CA USA), the antibodies were procured from BD Biosciences, San Jose, CA USA. 

*Surface Staining.* Freshly isolated PBMCs concentration was adjusted to 0.2 × 10^6^ per test and was resuspended in FACS buffer (PBS containing 1% FBS, 2 mM EDTA and 0.01% azide). FcR blocking reagent (Miltenyi Biotec GmbH, Germany) was added to the cells to block Fc receptors present on the surface of the lymphocytes. Surface antigens were labelled by incubating with monoclonal antibodies for 20 min at 4°C in dark followed by washing with PBS (0.5% BSA) and fixing with 1% paraformaldehyde.

*Intracellular Staining.* Fresh PBMCs (0.2 × 10^6^ per test) were resuspended in FACS buffer and were fixed by using BD Cytofix buffer for 30 min. After fixation, cells were washed and resuspended in FACS buffer. For intracellular labelling, following surface FcR receptor blocking, cells were incubated with Cytofix/Cytoperm buffer set (BD biosciences, USA) for 20 min at 4°C. All incubations were performed at 4°C in the dark. After fixation and permeabilisation, cells were incubated with monoclonal anti-human CTLA-4, IL10, and TGF-**β**_1_ antibodies in different tubes for 30 min at 4°C in dark. This was followed by washing the cells with PBS (0.5% BSA) and fixing with 1% paraformaldehyde.

Single color compensation was performed prior to acquisition of samples in the FACS Aria II. For each experiment, 50,000 events were acquired with appropriate isotype control and data were analyzed using FACS Diva software (Becton Dickinson, USA). Results are expressed as parent percentage of gated positive cell frequencies in mean ± SD.

### 2.7. RNA Extraction, cDNA Preparation, and Gene Expression Assays

Total cellular RNA was isolated from HEV rORF2p stimulated/control PBMCs frozen at −80°C using RiboPure RNA extraction kit (Life Technologies, CA USA). The integrity of RNA was checked and quantified (ND-1000, Nanodrop Technologies) [17]. The cDNA was synthesized from 1 *μ*g of total RNA using high capacity cDNA synthesis archive kit (Life Technologies, CA USA) following the manufacturer's instructions.

Based on real-time quantitative reverse transcription-polymerase chain reaction (RT-PCR), custom-made TaqMan Low Density Arrays (TLDA) were used for mRNA expression profiling in the unstimulated/HEV rORF2p stimulated PBMCs. These arrays contained predesigned primers and TaqMan probes (FAM reporter dye at the 5′ end of each TaqMan MGB probe and a nonfluorescent quencher at the 3′ end). Each array contained eight sample loading ports. A 2.5 *μ*L of each 20 *μ*L cDNA (~125 ng RNA equivalent) reaction was loaded into each port. These arrays contained primers and probes for 26 selected and 2 endogenous control genes (Table 2). Amplification and real-time analysis of cDNA samples loaded onto the TLDA were performed using an ABI-7900HT real-time PCR instrument. The results were analysed using SDS version 2.2 software (Life Technologies, CA USA).

Gene expression in unstimulated PBMCs was carried out in 33 AVH-E patients and 20 recovered individuals and the data was normalized with 33 healthy controls. Similarly, HEV rORF2p stimulated gene expression was carried out in 10 AVH-E, 10 recovered individuals, and 10 healthy controls. Normalization of the stimulated data was done against the unstimulated data of the respective groups.

Relative gene expression values were obtained employing comparative Ct method using relative quantification (RQ) Manager Software v1.2 (Applied Biosystems). GAPDH and 18sRNA were used as endogenous controls. RQ values of each study group were used to calculate mean.

### 2.8. Statistical Analysis

For all flow cytometry related data, BD FACS DIVA software was used and the data comparisons are expressed as mean ± SD. The peripheral marker frequency data of three study populations were analyzed using discriminant function analysis (DFA) [18]. For peripheral frequency analysis, statistically significant differences between groups were assessed using ANOVA with Bonferroni* post hoc *corrections. The differences were considered statistically significant at two-tailed *P* values < 0.05. All statistical analyses were performed with SPSS 20 software (SPSS Inc., Chicago, IL, USA). For all gene expression-related data, after normalized with respective controls, RQ values ≥ 2-fold were considered as significant up/downregulation of gene expression.

## 3. Results

### 3.1. Characteristics of the Study Subjects

There were no significant differences in the age and sex of the study population. ALT levels were significantly higher in AVH-E patients compared to the recovered individuals and controls. IgM levels were significantly higher and IgG levels were lower in AVH-E patients compared to the recovered individuals (Table 1) as reported [6–8].

### 3.2. Immunophenotyping of Unstimulated and Stimulated PBMCs

#### 3.2.1. Unstimulated PBMCs

**Figure 1:**
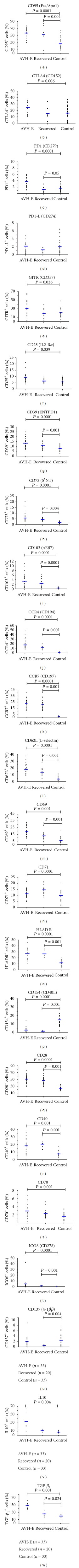
Peripheral expression profiles on the unstimulated PBMCs of hepatitis E patients by flow cytometry. Unstimulated PBMCs from controls, AVH-E, and recovered individual groups were stained with antibodies against different phenotype markers, apoptotic/inhibitory (a–f), ectonucleotidase (h-g), integrin (i), chemokine and selectin (j–l), activation (m–o), costimulatory (p–u), and Treg-associated cytokines (v-w). An isotype-matched antibody was used as a negative control. Each dot represents an individual data point and the horizontal lines represent the mean. The ANOVA with Bonferroni* post hoc *corrections was used to compare differences among groups. Data are representative of mean ± SD.

*AVH-E versus Control*: Significantly higher frequencies of CD95, CTLA-4, PD1, GITR, CD25, CD103, CCR4, CCR7, CD62L, CD73, CD39, HLA DR, CD69, ICOS, CD40, CD70, CD28, IL10, and TGF-*β*_1_ and lower frequency of CD154 in AVH-E patients.* Recovered versus Control*: Significantly higher frequencies of CD95, CD103, CCR4, CCR7, CD62L, CD73, CD39, HLA DR, CD69, CD71, ICOS, CD40, CD70, CD28, and TGF-**β**_1_ and lower frequencies of CD154, CD137 in the recovered individuals (Figure 1).

### 3.3. Stimulated PBMCs

**Figure 2:**
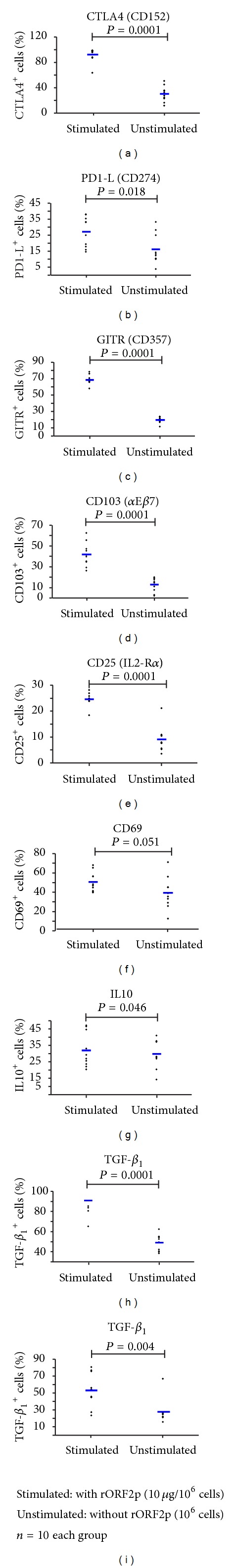
HEV rORF2p stimulated peripheral expression profiles in hepatitis E patients by flow cytometry. HEV rORF2p stimulated expression of inhibitory (CTLA-4, PD1L, and GITR), integrin (CD103), activation (CD25, CD69), and Treg-associated cytokines (IL10, TGF-**β**_1_) on PBMCs of AVH-E (a–h) and recovered (i);* n* = numbers of patients. Each dot represents an individual data point and the horizontal lines represent the mean. The ANOVA with Bonferroni* post hoc *corrections was used to compare differences among groups. Data are representative of mean ± SD.

AVH-E patients had higher frequencies of CTLA-4, PD1L, GITR, CD103, CD25, CD69, IL10, and TGF-**β**_1_ in rORF2p stimulated compared to the unstimulated PBMCs (Figures 2(a)–2(h)). Recovered individuals had higher frequencies of TGF-**β**_1_ only in rORF2p stimulated compared to the unstimulated PBMCs (Figure 2(i)). The percentages of all studied markers were comparable in HEV rORF2p stimulated versus unstimulated PBMCs of the control individuals (*P* > 0.05 in each). The frequencies of HEV rORF2p stimulated costimulatory molecules were not assessed in the study subjects due to nonavailability of the cells.

### 3.4. Discriminant Function Analysis (DFA)

**Figure 3:**
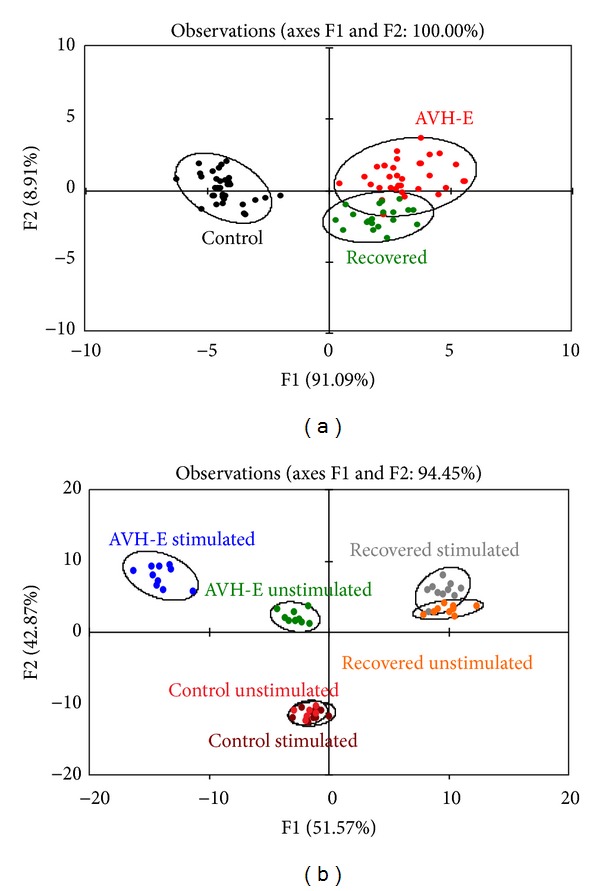
Clustering of markers on* naïve* and HEV rORF2p stimulated PBMCs of hepatitis E patients and healthy controls. A graphical representation of the discriminatory potential of discriminant function analysis (DFA). The analysis was used to select variables that maximally discriminate among the (a) discriminant function analyses of unstimulated PBMCs, AVH-E (red), recovered (green), and controls (black). Circles represent the 95% confidence eclipses. (b) Discriminant functions analysis of HEV rORF2p stimulated PBMCs. AVH-E stimulated (blue), AVH-E unstimulated (green), stimulated recovered (grey), unstimulated recovered (orange), stimulated controls (brown), and unstimulated controls (red). Circles represent the 95% confidence eclipses.

To improve the analysis of peripheral marker profiles the more discriminating FDA approach was performed. The principal component analysis (PCA) transformed data showed separate clusters for unstimulated PBMCs of the patient groups and control group (Pillai's trace = 0.0001, *P* = 0.0001; Figure 3(a)).

The HEV rORF2p stimulated PBMCs formed two distinct clusters only for AVH-E patient group; however, such two distinct clusters were not observed for recovered and control groups (Pillai's trace > 0.0001, *P* = 0.0001; Figure 3(b)) indicating a difference in peripheral expression pattern in acute phase of HEV infection.

### 3.5. Gene Expression Profiles of Unstimulated and Stimulated PBMCs

**Figure 4:**
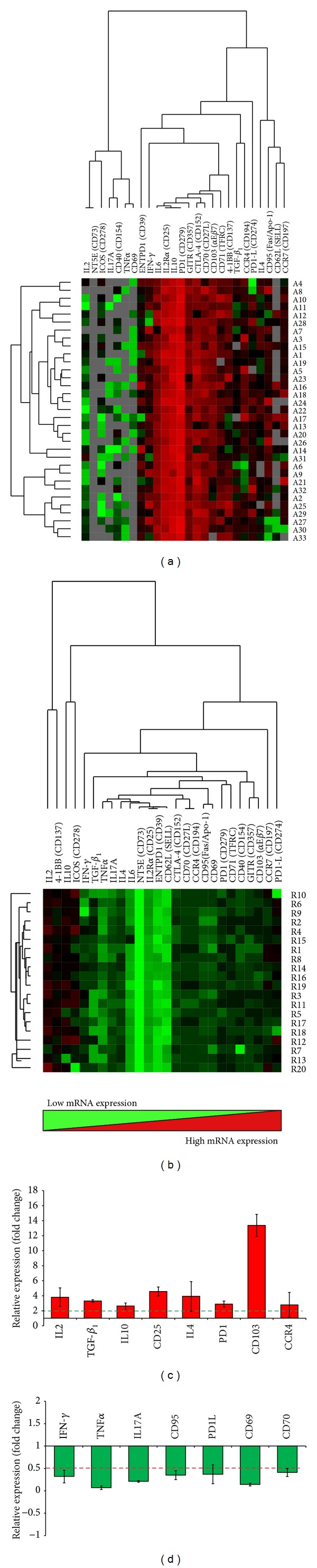
Gene expression profiles in hepatitis E infection by TLDA. (a) Gene expression profiles using customized TLDA panel by two-way hierarchical clustering on unstimulated PBMCs from AVH-E patients, recovered individuals after normalization with healthy controls. Each colored cell in the two hit maps represents the relative levels of expression of particular genes in a study subject. Green indicates low levels and red indicates high levels of gene expression. Values for 26 selected genes were hierarchically clustered on log transformation. The corresponding gene of each cluster is listed by a gene symbol on the top side of the images. Genes were ordered according to their cluster determined by the* k*-means algorithm. (a) AVH-E patients denoted as A1–A33; (b) recovered individuals denoted as R1–R20 on the right side of the images. Values increase from green to red, via black. (c-d) HEV rORF2p stimulated gene expression of* in vitro* cultured PBMCs of AVH-E patients. The values shown are the mean fold change of rORF2p stimulated PBMCs compared with the unstimulated PBMCs. Values are expressed as mean ± standard deviation.

In AVH-E patients IFN-*γ*, CD25, IL4, IL6, IL17A, CD95, CTLA-4, PD1, PD1L, GITR, CD39, CD70, CD137, CD71, CD103, CCR4, CCR7, IL10, and TGF-**β**_1_ genes were upregulated. Only CD73 gene was downregulated. In recovered individuals CD25, IL6, TNF*α*, CD39, CD73, CD40, and CD62L genes were downregulated in unstimulated PBMCs (Figure 4(a)) (Table 2).

In AVH-E patients IL2, CD25, IL4, PD1, CD103, CCR4, IL10, and TGF-**β**_1_ genes were upregulated (Figure 4(b)), while IFN-*γ*, TNF*α*, IL17A, CD95, PD1L, CD70, and CD69 genes were downregulated (Figure 4(c)). In recovered individuals only IL2 gene was upregulated in rORF2p stimulated PBMCs (Table 2).

## 4. Discussion

HEV infection is a dynamic process with most of the infected individuals recovering without sequelae. After entering through the oral route and inducing intestinal immunity, HEV recirculates through liver and arrives to the peripheral blood [19].

In an attempt to identify the key molecules involved in the pathogenesis of HEV infections, we have analyzed the expression profiles of activatory, inhibitory, integrin, homing, ectonucleotidase machinery, costimulatory, inflammatory markers, and Treg-associated cytokines on unstimulated and* in vitro *HEV rORF2p stimulated PBMCs of hepatitis E patients in the acute and recovered phases of illness.

Meager information is available regarding the peripheral phenotypic marker expressions in HEV patients. The outcome of HEV infection is reported to be dependent on both the T cell subsets and antigen nonspecific inflammatory cells recruited to the liver [12]. An expansion of the overall CD4^+^ cell population but no change in CD8^+^ cells in the peripheral blood of patients with acute hepatitis E patients [3], an increase in the proportion of CD8^+^ T cells in AVH-E but no change in the CD4^+^ T cell compartment in peripheral blood [19], unchanged percentages of CD4^+^ & CD8^+^ T cells in AVH-E and recovered individuals compared to the controls and significantly increased CD8^+^ T population in the recovered individuals compared to AVH-E reported by us clearly indicate the probable modulation of immune response by peripheral immune cells [7]. Further, acute hepatitis E infection is reported to be associated with a reversible alteration in the proportions and activation status of the peripheral NK/NKT cells and NK subsets [13]. Elevation of peripheral CD11c, HLA DR, CD11c/CD86, and HLADR/CD86 expressions in the acute hepatitis E patients with viral load has suggested involvement of these cells [14]. An increased expression of the CD11a integrin in naïve CD45RA^+^ T cells and overexpression of CCR5 and CCR9 during AVH-E infection have suggested an enhanced recruitment of these cells from periphery to the target tissue during the early phase of infection. An expansion of CD38^+^ CD69^+^ T cells in the acute phase compared to the resolving phase of infection with an increased in mRNA expression of IFN-gamma, TNF-*α* and IL-4 has reflected an increment in CD3^+^ CD38^+^ CD69^+^ T cells [19]. A decrease in CD4, an increase in CD8 cell counts, and lowered CD4/CD8 cell ratio in Indian pregnant women with fulminant hepatic failure E have been put forward as a plausible reason for severity of the disease [20].

Increased frequencies of CTLA-4, PD1, GITR, CD25, and IL10 in the studied AVH-E patients and no change in the unstimulated PBMCs expression profiles among the patients in acute and recovery phases of illness could be associated with HEV infection. Among 23 molecules assessed, frequencies of only TGF-*β*1 in the both patient's categories and CD25, CD69, CD103, PD1L, GITR, CTLA-4, and IL10 were altered in the HEV rORF2p stimulated PBMCs of acute patients. This could be due to inherent defect in the activation of T cells in HEV-infected individuals [3] and/or the inability of exogenous recombinant HEV ORF2 protein to process CD8 T cells. Hence, we might be capturing the molecules present on the CD4^+^ T cells only. This theory supports our previous report of higher expression of CTLA-4, PD1, GITR, CD95, CD103, and CD73 on the CD4^+^ T regulatory and T effector cells of HEV patients [9].

Higher expression of CTLA-4, PD1/PD1L in the unstimulated and HEV rORF2p stimulated PBMCs of the current study and previously reported higher frequency of the same on CD4^+^ Treg cells of acute hepatitis E patients might be involved in the suppression of T cell response [9]. Association of CTLA-4 and PD1 with the impairment of T cell response in chronic HEV patients supports our observation [21].

Higher expressions of CD103 indicating its involvement in homing of effector immune cells to sites of inflammation in AVH-E patients cannot be ruled out [22]. Consumption of IL2 by the Treg cells [23] could be attributed to the absence of IL2 at the protein level of the previous report [8]. Difference in scenario at the gene level for IFN-*γ*, IL17A, CD95, PD1L, and CD70 for unstimulated and HEV rORF2p stimulated PBMCs suggests that there may be lack of robust HEV rORF2p-specific peripheral response in the HEV-infected patients [7]. Higher IL2 expression in AVH E patients and lower IL2 expression in the recovered individuals & higher expression of IL 10 at the mRNA level of the current study does not match with the only reported gene expression study on HEV elucidating decreased IL-2 expression in both acute and resolving phases and reduced IL-10 expression in the resolving phase [19]. This discrepancy could be attributed to the difference in postonset days of illness of the patient population.

Reports of detection of nonreplicative HEV RNA in the PBMCs of acute hepatitis E patients by Ippagunta et al. and HEV RNA positivity in the sera after normalization of transaminases by Chandra et al. have indicated that PBMCs are not the site for HEV replication and that liver injury is independent of peripheral viral replication [24, 25].

Comparable and very low expressions of lineage negative DCs and different markers on the PBMCs among the self-limiting hepatitis E patients have been attributed to migration of these cells from periphery to lymphoid areas during HEV infection [26, 27]. In consideration of the above, further studies aiming to detect the key HEV-specific molecules on peripheral CD8^+^ T cells and in the site of infection, liver (known to harbor more CD8^+^ cells), are warranted that may provide an overall scenario.

Overall, our data elucidates distinct expression patterns of inhibitory, integrin, activatory, and Treg-associated cytokine genes complemented by their frequency data on the PBMCs of patients with self-limiting HEV infection, suggesting that these molecules could be the major players in the fine tuning of immune response in self-limiting hepatitis E infection.

## Figures and Tables

**Table 1:** Characteristics of study subjects.

Parameters	AVH-E	Recovered	Controls
Study population	*n* = 43	*n* = 30	*n* = 43
Age (Years)	28.18 ± 10.04	32.95 ± 14.41	30.80 ± 3.39
Sex ratio (M : F)	27 : 16	15 : 15	26 : 17
ALT (IU/L)	409.60 ± 374.78	28.45 ± 8.04	19.20 ± 6.56
IgM titre	10199.70 ± 8522.26	5880.0 ± 26591.20	Negative
IgG titre	28303.03 ± 19305.12	544880.0 ± 35619.62	Negative
Postonset days of illness (POD)	10.96 ± 5.07	84.75 ± 6.29	NA

Values are expressed in mean ± SD. *n*: number. NA: not applicable.

**Table 2:** Gene expression quantified using TaqMan Low Density Arrays and normalized with respective controls.

	AVH-E (*n* = 33)	Recovered (*n* = 20)	HEV-specific gene expression	
Gene ID and name	Fold change (Mean ± SD)		AVH-E (*n* = 10)	Recovered (*n* = 10)
			72 hrs	
Th_1_/anti-inflammatory cytokines				
IL2	1.48 ± 0.46	1.41 ± 0.64	3.8 ± 1.24	3.70 ± 1.81
IFN-*γ*	3.26 ± 1.05	0.62 ± 0.01	0.32 ± 0.14	0.53 ± 0.07
TGF*β*1	2.07 ± 0.00	0.51 ± 0.01	3.30 ± 0.16	1.37 ± 0.14
IL10	11.35 ± 0.30	1.15 ± 0.02	2.61 ± 0.43	0.58 ± 0.03
IL2R*α* (CD25)	8.96 ± 1.04	0.24 ± 0.05	4.56 ± 0.60	0.67 ± 0.02
Th_2_/proinflammatory cytokines				
IL4	2.11 ± 0.02	0.59 ± 0.01	3.91 ± 1.96	0.67 ± 0.07
IL6	4.97 ± 0.02	0.28 ± 0.01	1.99 ± 1.33	0.52 ± 0.04
TNF*α*	0.87 ± 0.01	0.34 ± 0.02	0.07 ± 0.04	0.51 ± 0.01
IL17A	8.03 ± 2.64	0.52 ± 0.05	0.21 ± 0.02	0.52 ± 0.03
Ectonucleotidase				
ENTPD1 (CD39)	2.77 ± 0.05	0.18 ± 0.01	0.59 ± 0.17	0.61 ± 0.03
NT5E (CD73)	0.18 ± 0.00	0.07 ± 0.01	1.66 ± 0.06	0.51 ± 0.18
Apoptotic/inhibitory				
Fas/Apo-1 (CD95)	3.07 ± 0.11	0.53 ± 0.02	0.35 ± 0.10	0.54 ± 0.04
CTLA-4 (CD152)	6.53 ± 0.22	0.59 ± 0.01	1.59 ± 0.99	0.91 ± 0.02
PD1 (CD279)	16.80 ± 0.08	0.68 ± 0.00	2.88 ± 0.42	0.53 ± 0.04
PD1-L (CD274)	2.80 ± 2.39	0.87 ± 0.09	0.37 ± 0.21	0.54 ± 0.07
GITR (CD357)	4.21 ± 0.07	0.63 ± 0.00	0.82 ± 0.40	0.62 ± 0.21
Costimulatory				
CD40 (CD154)	1.31 ± 0.12	0.61 ± 0.01	0.92 ± 0.29	0.61 ± 0.05
CD70 (CD27L)	4.90 ± 0.19	0.64 ± 0.00	0.40 ± 0.09	0.58 ± 0.01
ICOS (CD278)	1.35 ± 0.23	1.10 ± 0.05	1.81 ± 0.56	0.56 ± 0.23
4-1BB (CD137)	3.56 ± 0.02	1.07 ± 0.03	1.22 ± 0.78	0.56 ± 0.11
Activation molecules				
CD69	1.00 ± 0.02	0.51 ± 0.02	0.14 ± 0.03	0.55 ± 0.01
TFRC (CD71)	3.55 ± 0.10	0.70 ± 0.01	0.96 ± 0.34	0.50 ± 0.02
Chemokine and homing receptors				
SELL (CD62L)	1.14 ± 0.06	0.19 ± 0.01	0.58 ± 0.03	0.51 ± 0.04
*α*E*β*7 (CD103)	4.10 ± 0.38	0.68 ± 0.10	13.37 ± 1.47	0.57 ± 0.01
CCR4 (CD194)	3.07 ± 0.13	0.64 ± 0.01	2.78 ± 1.65	0.52 ± 0.05
CCR7 (CD197)	2.67 ± 0.14	0.84 ± 0.01	0.74 ± 0.15	0.73 ± 0.02

In unstimulated PBMCs of AVH-E and recovered individuals, mean fold changes are compared after normalization with healthy control data. HEV rORF2p stimulated *in  vitro* cultured PBMCs gene expression pattern after normalization with *in  vitro* cultured unstimulated PBMCs from respective groups (bold). The values shown are the mean fold change compared with unstimulated/stimulated cells of respective groups and are expressed in mean ± standard deviation.

## Abbreviations

AVH-E: acute viral hepatitis E
HEV: hepatitis E virus
ALT: alanine transaminase
PBMCs: peripheral blood mononuclear cells
HCV: hepatitis C virus
HBV: hepatitis B virus
CTLA-4: cytotoxic T lymphocyte antigen 4
TGF-*β*_1_: transforming growth factor beta 1
IL10: interleukin 10.
